# Unusual Presentation of Subcutaneous Panniculitis‐Like T‐Cell Lymphoma

**DOI:** 10.1002/ccr3.71572

**Published:** 2025-12-03

**Authors:** Mona Alkallabi, Hend Alotaibi, Khalid Nabil Nagshabandi, Reem Bin Idris, Amal H. Abualola, Naif Ahmed Alshehri, Ahmed Alhumidi

**Affiliations:** ^1^ Department of Dermatology, College of Medicine King Saud University Riyadh Saudi Arabia; ^2^ Department of Dermatology King Fahad Medical City Riyadh Saudi Arabia; ^3^ Department of Pathology, College of Medicine King Saud University Riyadh Saudi Arabia

**Keywords:** atypical presentation, cutaneous lymphoma, subcutaneous panniculitis‐like T‐cell lymphoma, ulcerated plaques

## Abstract

Subcutaneous panniculitis‐like T‐cell lymphoma (SPTCL) is a rare variant of primary cutaneous T‐cell lymphoma primarily affecting subcutaneous adipose tissue and mimicking lobular panniculitis. It often presents with erythematous subcutaneous nodules or plaques, and diagnosis can be challenging due to its diverse clinical manifestations. We report an unusual presentation of SPTCL in a 40‐year‐old female with recurrent, painless ulcerated plaques on the thighs and lower legs, accompanied by systemic symptoms including weight loss, fatigue, and laboratory abnormalities. Initially misdiagnosed as erythema nodosum, the patient was treated with antibiotics and corticosteroids without significant improvement. Histopathology and immunohistochemistry from skin biopsies revealed atypical lymphocytic infiltration consistent with SPTCL (CD3+, CD30+, CD4+, CD8−, CD56−). This case adds to the spectrum of SPTCL presentations, emphasizing the importance of considering SPTCL in panniculitis‐like lesions refractory to conventional treatment. Prompt histopathological evaluation with immunophenotyping is crucial for early diagnosis and appropriate management to improve patient outcomes.

## Key Clinical Message

Subcutaneous panniculitis‐like T‐cell lymphoma may present as ulcerated plaques mimicking common inflammatory conditions. Early histopathologic and immunophenotypic evaluation is essential in refractory panniculitis‐like lesions to ensure accurate diagnosis and appropriate management.

## 1 Introduction

Subcutaneous panniculitis‐like T‐cell lymphoma (SPTCL), first described by Gonzalez et al. in 1991, is a rare form of primary cutaneous T‐cell lymphoma that primarily affects the subcutaneous adipose tissue and accounts for nearly < 1% of all cutaneous lymphomas. It is a cytotoxic T‐cell lymphoma that causes pleomorphic T cells and macrophages to infiltrate the subcutaneous tissue, and mimic lobular panniculitis. Clinically characterized as tender, erythematous subcutaneous nodules or plaques over the trunk and extremities mimicking a lipoma, it is often accompanied by systemic symptoms such as fever, weight loss, and malaise [1]. Histologically, SPTCL is characterized by lobular panniculitis with a prominent subcutaneous infiltration of atypical lymphocytes showing adipotropism rimming individual fat cells and lobules [1, 2]. The disease primarily affects young women, with 20% of instances involving patients under the age of 20 [3]. The World Health Organization‐European Organization for Research and Treatment of Cancer (WHO‐EORTC) classification of cutaneous lymphomas in 2005, defined SPTCL classified into two main types based on the phenotype of T‐cell receptor and immunohistochemistry. CD8+ cytotoxic αβ T‐cell lymphoma (SPTL‐AB) with a (CD4−, CD8+, and CD56−) phenotype, is restricted to subcutaneous fat and has an indolent and persistent clinical course. Cutaneous γδ T‐cell lymphoma (CGD‐TCL), which is characterized by a unique phenotype (CD4−, CD8−, CD56+) has a dismal prognosis [3, 4]. In 20% of cases, SPTCL complicates hemophagocytic lymphohistiocytosis (HLH), an uncommon and possibly lethal condition that causes immunological hyperactivation and inflammation, resulting in multiple organ failure [1, 2, 3].

SPTCL is characterized by rare diverse clinical presentations, making diagnosis challenging. Previous reported instances showed multiple presentations such as: erythema nodosum‐like, eschar‐like crusting, without erythema or tumors, mimicking Adult‐onset Still's Disease, vacuolar interface dermatitis resembling lupus erythematosus panniculitis, right thigh hypertrophy, necrosis and ulceration, and venous stasis ulceration [3, 5, 6, 7, 8, 9, 10, 11, 12]. We report a rare and unusual case of subcutaneous panniculitis‐like T‐cell lymphoma presenting clinically as ulcerated plaques.

## 2 Case History/Examination

**FIGURE 1:**
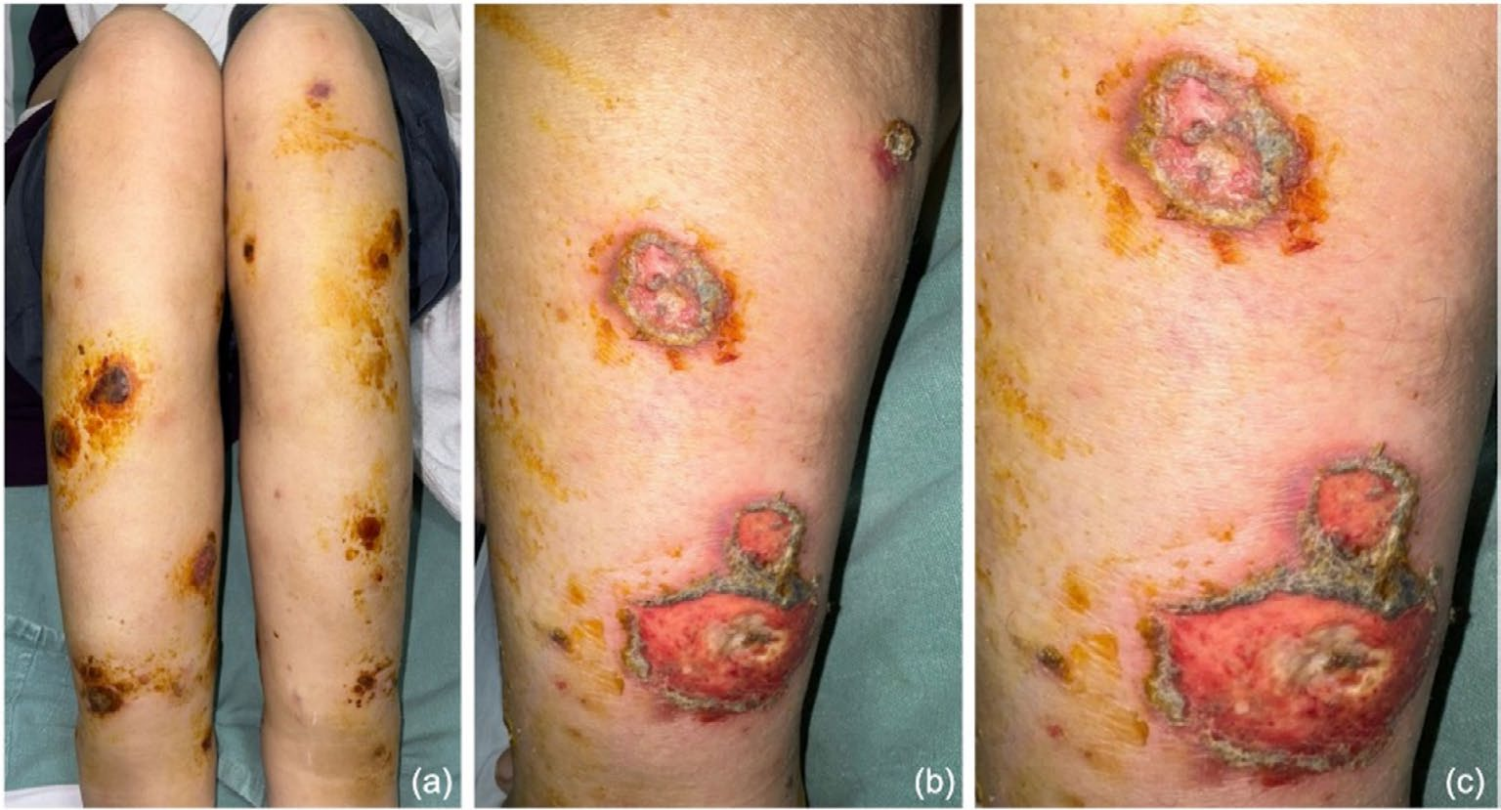
(a, b, c) Multiple well defined, erythematous, indurated, subcutaneous nodules. Some of them are secondarily ulcerated with erythematous granulation tissue and yellowish to brown crustations on top. They are non tender with minimal exudate and normal surrounding tissue on bilateral lower limbs. Peripheral pulses were palpable and peripheral sensation was intact. Positive palpable lymph node measuring 2 × 2 cm in the inguinal area.

A 40‐year‐old female, with no particular medical or surgical history, presented to the emergency department at our university hospital with a 4‐month history of painless recurrent ulcers and erosions on the thighs and lower legs. She had generalized fatigue, a poor appetite, and lost 10 kg over 4 months. Prior to the onset, she had a flu‐like illness, which resolved in 1 week and was followed by a single red asymptomatic patch on the medial knee 3 weeks later. Over the course of a month, the lesions spread to involve the thighs and lower legs bilaterally. They started as a progressive red skin eruption, which then discharged blood and serous fluid. She complained of lower limb edema up to the thighs and weakness for 2 months. She had no fever or other systemic symptoms. On physical examination, the patient looked ill and fatigued with tachycardia. Multiple well‐defined ulcerated plaques, which varied in size and had active borders, were present over the thighs and lower legs bilaterally. some of which were covered with hemorrhagic, yellow, and black crusts. The lesions were accompanied by lower‐limb swelling (Figure 1). No lesions were noted in the mucous membranes, scalp, or nails. There was no organomegaly; however, left‐inguinal lymph node enlargement was present.

## 3 Differential Diagnosis, Investigations and Treatment

Prior to being admitted, she initially presented to a private hospital and was diagnosed with Erythema Nodosum. She was given Augmentin for 14 days with limited improvement. She was then prescribed Dexamethasone and Ertapenem for 10 days, which showed a 50% improvement in her skin lesions. When she presented to us, she was taking oral Ciprofloxacin and Doxycycline, in addition to topical antibiotics, for 20 days with a moderate response. Laboratory results upon admission showed leukopenia (WBC 1.8 × 10^9^/L), macrocytic anemia (Hb 82 g/L, MCV 95.1 fL, retic count% 7.08%), high LFT (ALT 78 unit/L, AST 226.4 unit/L), high LDH (890 unit/L), and high D‐dimer (1.72 mcg/mL). She had positive readings for ANA (1:160), C4 complement (0.510), CCP (233.34), RF (22.30), SS‐A Ab (67.94), and TB quantiferon. A peripheral blood smear showed RBCs with a dimorphic blood picture, a few ovalocytes, teardrop poikilocytes, and mild polychromia. WBCs showed leukopenia, mild neutropenia, and a few reactive‐looking lymphocytes. The bone‐marrow study was normal. Imaging studies were comprehensive. A contrast‐enhanced CT scan of the chest was unremarkable, showing no thoracic malignancy or lymphadenopathy. However, a CT of the abdomen and pelvis revealed multiple well‐defined hypodense uterine lesions consistent with leiomyomas, along with a few nonspecific enlarged abdominopelvic lymph nodes. Additionally, mild to moderate ascites and subcutaneous edema were observed. A thyroid ultrasound demonstrated multiple bilateral thyroid nodules, with the largest measuring 1.2 × 1.2 cm, classified as TI‐RADS 3, without significant cervical lymphadenopathy. An ultrasound of the inguinal region revealed multiple suspicious lymph nodes on the left side, showing loss of fatty hilum, increased vascularity, and surrounding fat stranding, with the largest nodes measuring 2 × 2 cm and 2.6 × 1.2 cm. The right inguinal nodes were less concerning, retaining their fatty hilum, with the largest measuring 2.5 × 1 cm. A brain MRI was also performed, revealing no significant intracranial abnormalities.

**FIGURE 2:**
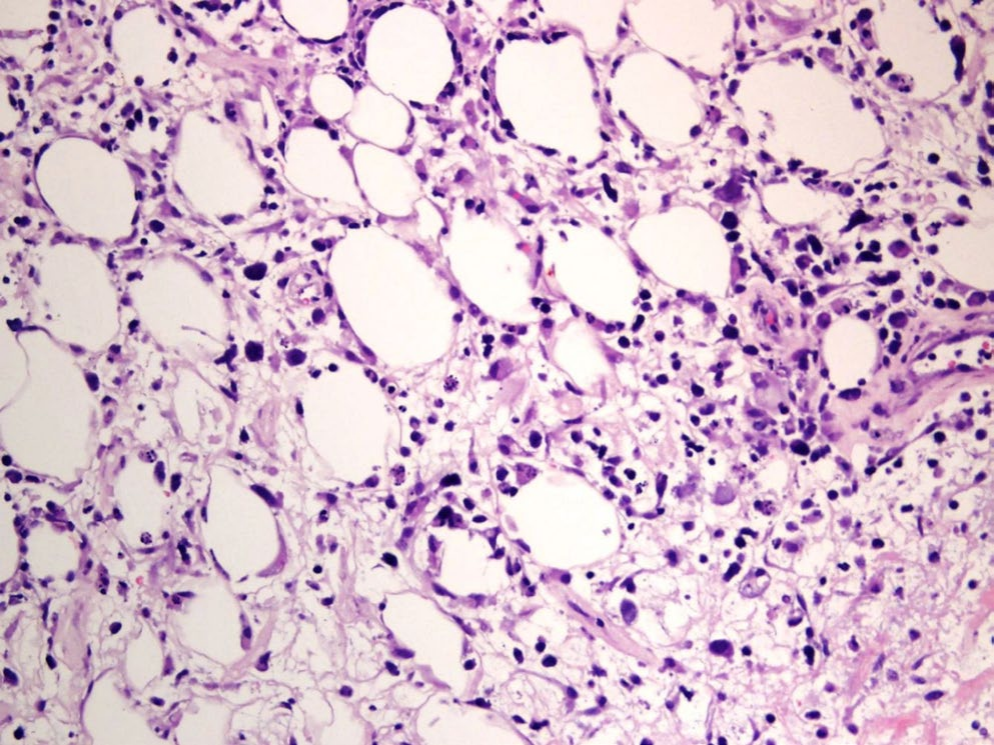
A photomicrograph of skin punch biopsy reveals subcutaneous infiltration of large atypical cells/lymphocytes (H&E stain, original magnification ×400).

**FIGURE 3:**
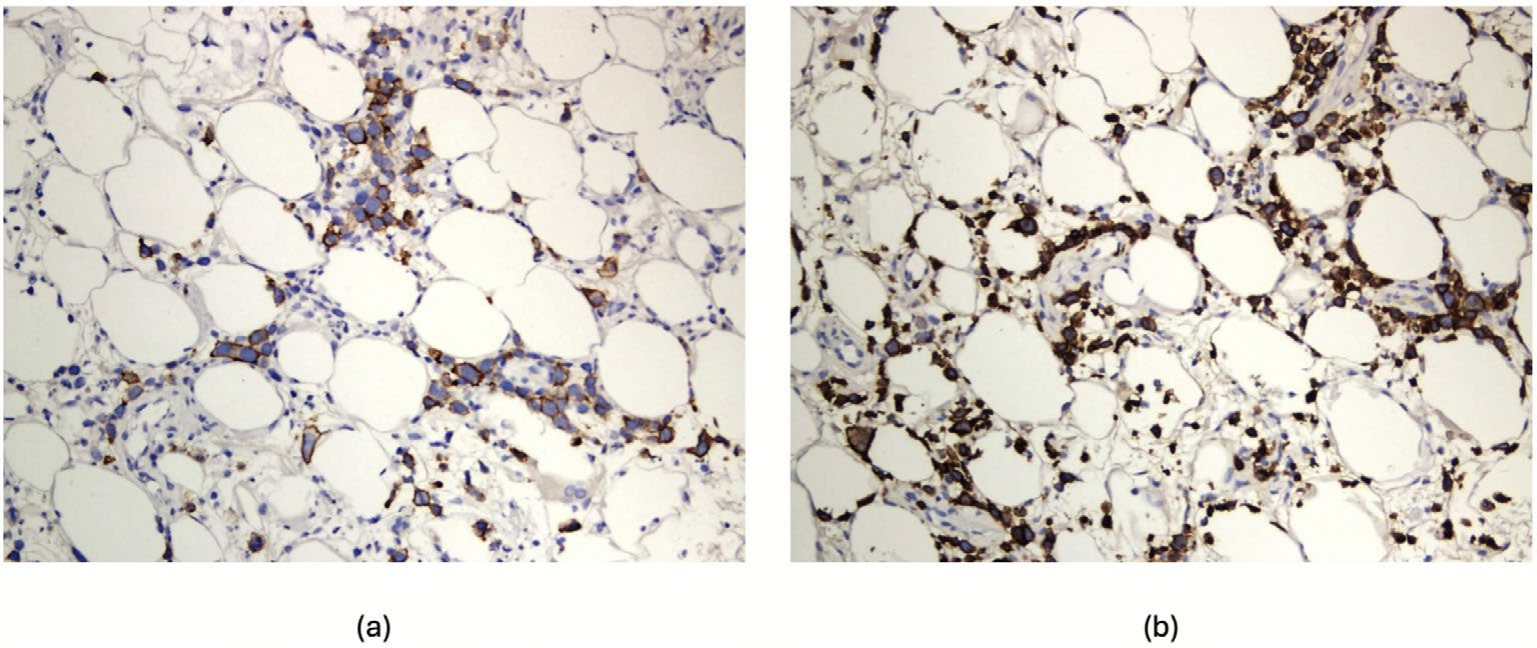
(a) CD3 immunohistochemistry stain is positive in the atypical cells. (Immunohistochemistry stain, original magnification ×400). (b) CD30 immunohistochemistry stain is positive in the atypical cells (Immunohistochemistry stain, original magnification ×400).

Histopathology from the skin biopsies revealed sloughing with crust formation in sections of the dermis. Granulation tissue with perivascular inflammation was seen in the papillary dermis. Multiple scattered atypical lymphocytes were found in the deep dermis and subcutaneous tissue (Figure 2). Immunohistochemistry analysis showed that the T‐cells were positive for CD3 (Figure 3a), CD30 (Figure 3b), CD2, CD4, and focally for Granzyme‐B, while negative for CD5, CD7, CD8, CD20, ALK1, and MPO. Based on the clinical presentation, histopathological findings, immunohistochemistry profile, and the exclusion of other potential causes, the patient was diagnosed with subcutaneous panniculitis‐like T‐cell lymphoma. The case was reviewed in a multidisciplinary meeting with dermatology, hematology, rheumatology, and infectious diseases specialists. Given the positive QuantiFERON‐TB result, the patient was started on antitubercular therapy with isoniazid and pyridoxine. The management plan included tapering her systemic corticosteroids, which she had been using since her treatment at the private hospital, to assess her skin's response without immunosuppression. The hematology team continued her evaluation, and a PET‐CT scan was scheduled to further assess systemic lymphoma involvement.

## 4 Conclusion and Results (Outcome and Follow‐Up)

The patient showed gradual improvement in her skin lesions with continued wound care and supportive management. Her systemic symptoms, including fatigue and appetite, improved, and her liver enzyme levels began to normalize. However, her left inguinal lymph node remained palpable. As of the time of discharge, she was alive and in stable condition under the care of the hematology‐oncology team. She was scheduled for close outpatient follow‐up with a comprehensive plan that included PET‐CT imaging and possible lymph node excision biopsy.

## 5 Discussion

Subcutaneous panniculitis‐like T‐cell lymphoma (SPTCL) is a rare variant of primary cutaneous T‐cell lymphoma that primarily affects the subcutaneous adipose tissue, mimicking lobular panniculitis [1, 2]. Epidemiological studies indicate a higher prevalence in females, with the exact etiology for this gender disparity remaining unclear. It may be linked to immunogenetic factors or hormonal influences on T‐cell function [13]. SPTCL generally follows an indolent clinical course, but approximately 20% of cases progress to hemophagocytic lymphohistiocytosis (HLH), a life‐threatening systemic inflammatory syndrome characterized by excessive immune activation. The development of HLH is associated with poor prognosis, increasing the risk of multi‐organ failure and mortality [13]. This underscores the need for long‐term monitoring in SPTCL patients to identify early signs of systemic progression.

The rarity and clinical heterogeneity of SPTCL often result in delayed diagnosis or misdiagnosis, as seen in our case [14]. The typical presentation consists of erythematous subcutaneous nodules or plaques; however, ulceration is uncommon and may correlate with a more aggressive disease course [15]. Our patient initially received an incorrect diagnosis of erythema nodosum and was treated with antibiotics and corticosteroids before the correct diagnosis was established. This highlights the diagnostic challenges of SPTCL and the crucial role of histopathology and immunohistochemistry in confirming the diagnosis [16].

Histopathological examination in our case showed lobular panniculitis with atypical lymphocytes infiltrating the subcutaneous tissue and adipotropism, confirming SPTCL of the αβ T‐cell subtype (CD3+, CD30+, CD4+, CD8−, CD56−). While CD8 positivity is typically associated with an indolent course, our patient's CD8‐negative phenotype with CD30 positivity suggests a less common but recognized variant with potential systemic involvement [17, 18]. Laboratory abnormalities, including leukopenia, elevated liver enzymes, and positive autoantibodies, raised suspicion of early HLH, though the patient did not exhibit classic HLH features such as fever or splenomegaly [19].

The clinical spectrum of SPTCL is diverse, with ulceration being a rare but previously reported finding. Kim et al. [20] documented a case initially misdiagnosed as erythema nodosum, similar to our patient, while Liu et al. [21] described SPTCL mimicking venous stasis ulceration [20, 21, 22]. These cases further emphasize the misleading nature of SPTCL presentations and the importance of early biopsy and immunophenotyping in differentiating it from other panniculitic conditions. The immunohistochemical profiles in SPTCL are crucial for both diagnosis and prognosis. The more common phenotype of CD4−, CD8+, and CD56− generally predicts an indolent clinical course [20]. However, our patient's phenotype, CD3+, CD30+, CD4+, CD8−, and CD56−, though less frequent, is associated with a chronic disease progression with systemic involvement. The coexpression of CD30 and CD4, with the lack of expression of CD8, points to less typical but still reported variants of SPTCL [21]. Systemic symptoms, such as those seen in our patient, indicate a more severe disease course that needs cautious long‐term follow‐up [19]. Previous cases reporting similar systemic features often involved aggressive disease, although our patient's response to immunosuppressive therapy indicates a relatively indolent clinical trajectory [23].

SPTCL treatment is individualized, with immunosuppressive therapy (e.g., corticosteroids, cyclosporine, methotrexate) being the first‐line approach. More aggressive or refractory cases may require chemotherapy or hematopoietic stem cell transplantation [4]. Our patient showed partial improvement with corticosteroids and antibiotics, suggesting a relatively indolent disease course, but continued monitoring, including PET scans, remains essential to assess ongoing disease activity and detect potential progression to HLH [19].

Recent genomic and immunobiology work has sharpened our understanding of SPTCL; germline loss‐of‐function variants in HAVCR2 (TIM‐3) are seen in a meaningful subset, especially in patients with HLH, linking defective TIM‐3 signaling to unchecked immune activation [24, 25]. Mechanistically, TIM‐3 restrains nucleic‐acid–triggered pathways; loss or blockade can amplify cGAS–STING/TLR signaling and downstream cytokines, offering a plausible bridge between genotype and the hyperinflammatory phenotype in severe cases [26]. Although we did not perform HAVCR2 testing, it may be most informative when HLH features or early‐age onset are present [25].

This case adds to the growing spectrum of SPTCL presentations, illustrating an unusual ulcerated manifestation with systemic involvement and an atypical immunophenotype. It reinforces the importance of early biopsy and immunophenotypic analysis for accurate diagnosis and timely management. Given the potential for HLH development and other complications, multidisciplinary management and close follow‐up are imperative to optimize patient outcomes. A side‐by‐side comparison of classic subcutaneous panniculitis‐like T‐cell lymphoma (SPTCL) features and our patient's presentation (Table 1) highlights key points.

**TABLE 1:** Typical presentation of subcutaneous panniculitis‐like T‐cell lymphoma (SPTCL) versus the current case.

Aspect	Typical SPTCL (Literature)	Current case
Skin lesions	Multiple subcutaneous nodules or indurated plaques in the fat, most often on the extremities. The overlying skin is usually intact. Ulceration is reported but uncommon [2, 20, 21, 22]	Multiple ulcerated plaques with hemorrhagic/yellow‐black crusts on both thighs and lower legs were present at first presentation
Pain/tenderness	Described as subcutaneous nodules that may be painful. When ulceration is present in reported cases, it is often inflamed and painful [16, 20, 21, 22]	Lesions were largely painless despite frank ulceration and necrosis
Distribution	Usually multifocal on the extremities (thighs, legs, arms) and/or trunk [2]	Bilateral thighs and lower legs with associated lower limb edema. No trunk, facial, or mucosal involvement
Systemic symptoms	Constitutional “B symptoms” such as fever, chills, malaise, anorexia, fatigue, and weight loss are common; many patients are febrile at presentation [2, 13, 14, 19]	Marked fatigue, anorexia, and ~10‐kg unintentional weight loss over 4 months, but no fever
Laboratory/HLH‐related changes	Cytopenias, elevated LDH, and elevated liver enzymes are frequent. A significant minority (15%–25% overall) develop hemophagocytic lymphohistiocytosis (HLH), often with high fevers and aggressive inflammation [2, 13, 14, 19]	Leukopenia, macrocytic anemia, ↑LDH, ↑transaminases, ↑D‐dimer, and inguinal lymphadenopathy raised concern for evolving HLH, but without fever or splenomegaly at that time
Initial working diagnosis	Frequently misdiagnosed as erythema nodosum, cellulitis, panniculitis, or “infected ulcer,” leading to courses of antibiotics or steroids before lymphoma is suspected [16, 20, 21, 22]	Initially managed as presumed erythema nodosum/infected ulcers with multiple antibiotics and steroids before biopsy confirmed SPTCL
Immunophenotype	Classic cytotoxic α/β T‐cell phenotype: CD3+, CD8+, CD4–, CD56–, with cytotoxic granule markers (TIA‐1, granzyme B, perforin). CD30 is usually negative [2, 4, 15, 18]	CD3+, CD4+, CD8–, CD30+, CD56– with focal granzyme B. CD4+/CD8– variants are rare, and diffuse CD30 expression is not typical for classic SPTCL

Subcutaneous panniculitis‐like T‐cell lymphoma is a challenging diagnosis due to its rare and diverse clinical presentations. This case highlights a unique presentation of SPTCL with ulcerated plaques and systemic symptoms, initially misdiagnosed as erythema nodosum. Clinicians should consider SPTCL in the differential diagnosis of panniculitis‐like lesions that are refractory to conventional treatment to ensure timely and appropriate management.

## Author Contributions

**Mona Alkallabi:** conceptualization, project administration, supervision, validation. **Hend Alotaibi:** conceptualization, project administration, supervision, validation. **Khalid Nabil Nagshabandi:** investigation, methodology, visualization, writing – original draft, writing – review and editing. **Reem Bin Idris:** methodology, resources, visualization, writing – original draft. **Amal H. Abualola:** methodology, resources, writing – original draft. **Naif Ahmed Alshehri:** visualization, writing – original draft, writing – review and editing. **Ahmed Alhumidi:** supervision, validation, visualization.

## Funding

The authors have nothing to report.

## Ethics Statement

The authors have nothing to report.

## Consent

Written informed consent was obtained from the patient for publication of the details of their medical case and any accompanying images.

## Conflicts of Interest

The authors declare no conflicts of interest.

## Data Availability

All data generated or analyzed during this study are included in this article. Further enquiries can be directed to the corresponding author.
